# Using resorbable extracellular bio-matrix for construction and reconstruction of hemodialysis vascular access 

**DOI:** 10.5414/CNP104S09

**Published:** 2025-11-28

**Authors:** Nina Keršnik, Tjaša Furlan, Jernej Vrtek, Zvezdana Dolenc Stražar, Boštjan Leskovar

**Affiliations:** 1 Vascular Disease and Vascular Access Unit, Trbovlje General Hospital, Trbovlje, and; 2 Institute of Pathology, Faculty of Medicine, University of Ljubljana, Ljubljana, Slovenia

**Keywords:** extracellular bio-matrix, arteriovenous fistula, vascular access surgery

## Abstract

Introduction: We assessed the use of resorbable extracellular bio-matrix in vascular access surgery. Materials and methods: A retrospective analysis was made of procedures where a resorbable extracellular bio-matrix was used for the construction or reconstruction of an arteriovenous (AV) fistula. A tubular shape of a certain length and diameter was made from sheets of resorbable bio-matrix (CorMatrix), which was then incorporated into AV anastomosis. Puncturing for hemodialysis began 8 – 10 weeks after construction. Results: Since 2016, our unit has used resorbable extracellular bio-matrix in 22 patients. We used it as an arterial circuit to the AV fistula vein, a reduction segment in high-flow AV fistulas, to extend the puncture area, as a connecting segment after partial removal of an AV fistula, and to construct the entire AV fistula instead of a graft. All procedures were successful with no significant peri- or post-operative complications. Additional procedures to ensure patency were also successfully performed. Histopathological analysis showed complete vascular differentiation of the bio-matrix resulting in thickening of the wall and narrowing of the lumen. Conclusion: The use of resorbable extracellular bio-matrix in vascular surgery is safe and feasible. It is recommended to use wider grafts to avoid thrombosis due to narrowing of the lumen. Further research and pre-prepared tubular bio-grafts of different diameters and lengths are needed.

## Introduction 

Arteriovenous fistulas (AVFs) are considered superior to arteriovenous grafts (AVGs) because of longer patency and lower complication rates [1]. An AVG is a possible alternative when an AVF cannot be constructed [2]. The most commonly used AVGs are synthetic, made from polytetrafluoroethylene (PTFE), but in recent years, several biological materials have been introduced in vascular access surgery [3, 4]. 

The acellular extracellular bio-matrix scaffold, derived from porcine small intestine submucosa (CorMatrix Cardiovascular, Inc., Roswell, GA, USA), has been widely used in cardiac and vascular surgery [5, 6, 7, 8]. Experience with CorMatrix in vascular access surgery is sparse and mostly limited to case reports and small case series [9, 10, 11]. This bio-matrix scaffold allows cells to migrate from adjacent tissues [12]. In only a few weeks, the bio-matrix degrades, leaving only autologous tissue behind. In this study, we present our results using CorMatrix for hemodialysis vascular access surgery. 

## Materials and methods 

In our prospective pilot case study, we analyzed operative procedures, perioperative complications, and long-term outcomes using an acellular extracellular bio-matrix scaffold (CorMatrix) for vascular access construction and reconstruction. All procedures were performed at the Vascular Disease and Vascular Access Unit, Trbovlje General Hospital, Trbovlje, Slovenia, between May 2016 and May 2024. 

All patients were older than 18 years and were kidney transplant candidates (since any foreign material is undesirable in these patients). Our exclusion criteria were age less than 18, pregnancy, and heart failure with a left ventricular ejection fraction of less than 35%. 

The Medical Ethics Committee approved the study (EK01/2016). 

### Operative technique 

All patients underwent complete clinical and ultrasound examination prior to enrollment in the study. All procedures were performed under regional or general anesthesia in the operating room. Patients were administered standard antibiotic prophylaxis prior to the procedure. After preparing the arterial and venous anastomosis, we determined the appropriate length of the bio-graft. We used 4-layer sheets of CorMatrix with a surface area of 4 × 7 cm or 7 × 10 cm and sutured them into a tubular graft of the desired length (20 – 30 cm). In the first few cases, we sutured tubular grafts with a 6 mm diameter, but due to marked hyperplasia of the endothelium, which resulted in bio-graft stenosis, we later switched to 8 mm diameter grafts. We started puncturing the bio-grafts 8 – 10 weeks after construction. In the case of vascular access stenosis or dysfunction, all the regular reconstruction techniques were used to preserve or re-establish patency. All percutaneous interventions were done under ultrasound guidance, which is the standard of care at our vascular access center. 

### Follow-up 

All patients had a morphologic ultrasound examination with measurement of the brachial artery flow before discharge and regularly at outpatient clinic visits. The first follow-up examination was performed 4 – 6 weeks after hospital discharge and every 3 months thereafter. 

### Statistical analysis 

Demographic and outcome variables were collected and analyzed with STATA, version 17.0 (StataCorp LLC, College Station, TX, USA). Continuous variables are reported as the median with range, and categorical variables as percentages. 

## Results 

Between May 2016 and May 2024, we used resorbable extracellular bio-matrix in 22 patients (median age 58.5 years (range 28 – 75), 68% male). All interventions were successful, with no significant peri- or postoperative complications (no considerable postoperative arm swelling, rupture, or infection of the bio-graft). 

We used CorMatrix as an arterial circuit to the fistula vein (proximalization of arteriovenous anastomosis PAVA), revision using distal inflow (RUDI)), a reduction segment in high-flow fistulas, to extend the puncture area, as a connecting segment after partial venous resection, and for the construction of the entire AVG instead of a PTFE graft. Detailed information on surgical procedures is shown in Table 1. 

We safely punctured the bio-graft 8 – 10 weeks after the operation. Hemostasis after needle removal was good, and we observed no significant hematomas. Puncturing a resorbable extracellular bio-matrix was more similar to puncturing a native vein than a PTFE graft. 

We successfully performed all additional procedures to ensure patency (thrombendarteriectomy, percutaneous transluminal angioplasty with/without stenting, and surgical reanastomosis). 

**Figure 1:**
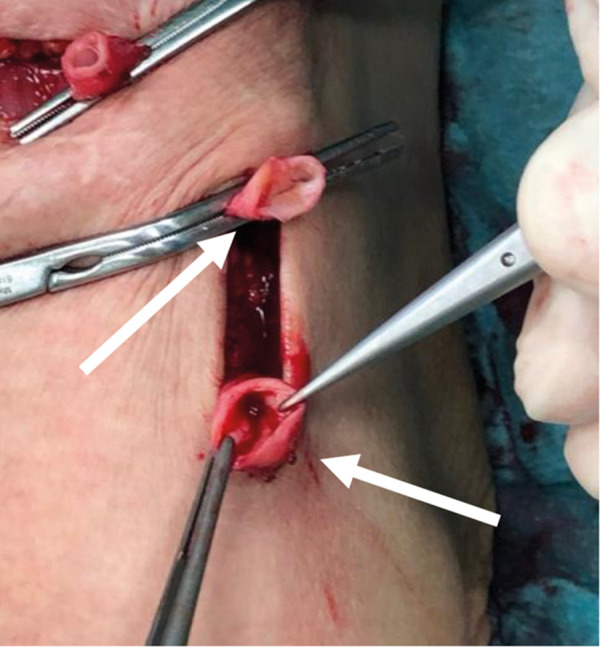
CorMatrix graft before explantation. Top arrow: venous part of the graft. Bottom arrow: arterial part of the graft with muscle wall.

**Figure 2:**
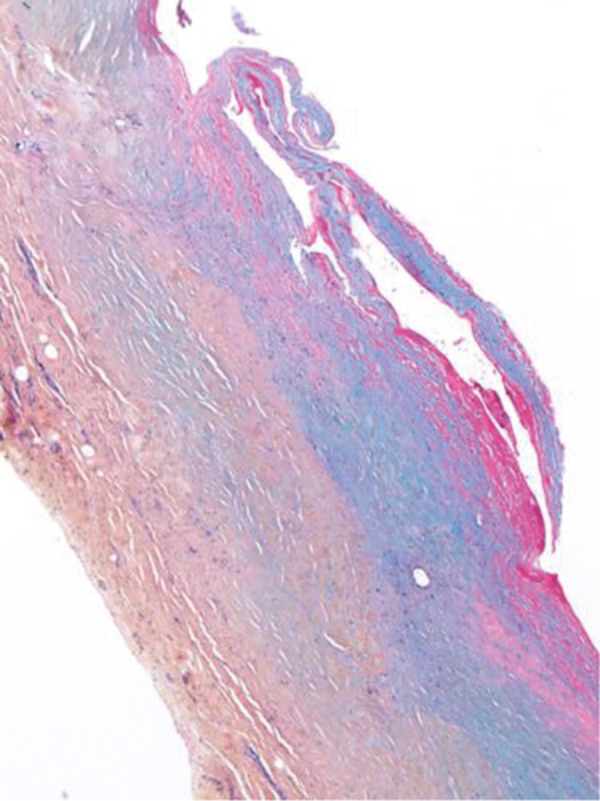
Cross-section of the vascular wall (×40). Blue: connective tissue in the inner part of the wall. Yellow: old connective tissue in the outer part of the wall.

**Figure 3:**
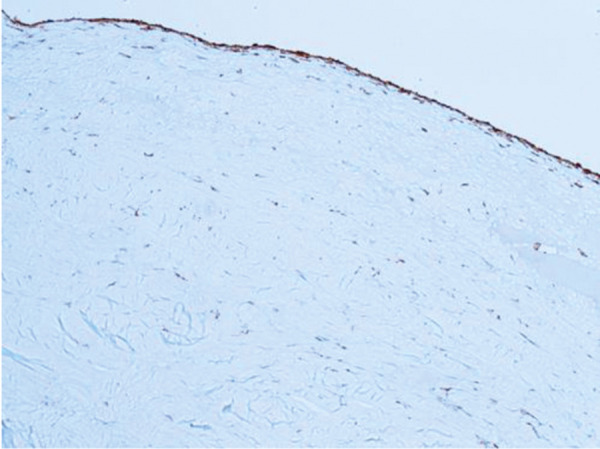
CD31 staining × 100: brown line – endothelium.

**Figure 4:**
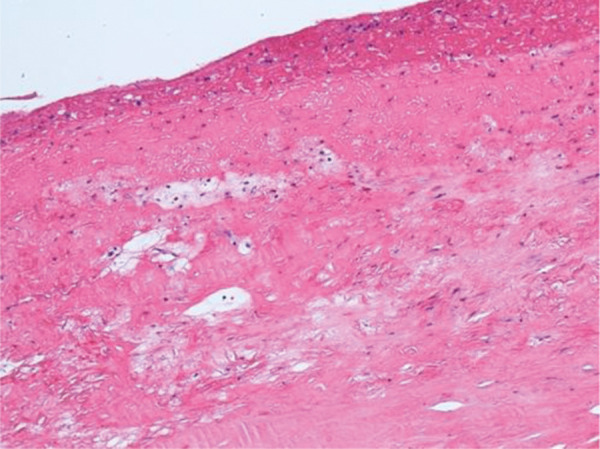
Hematoxylin and eosin staining × 100: foamy macrophages, the onset of atherosclerosis.

In 1 case, we observed repeated vascular access thrombosis, which resulted from pronounced bilateral hyperplasia of the endothelium and adventitia due to a narrow tubular CorMatrix graft (6 mm). After the closure of the AVG and partial explantation of the graft, part of the biological material was sent for histopathological examination (Figure 1). The pathologist described only native connective tissue with individual smooth muscle fibers (Figure 2), and endothelization of the inner stratum (evidence of the CD31 molecule, a marker for vascular differentiation (Figure 3)). Histopathological examination even showed foamy macrophages, a sign of the onset of atherosclerosis, in the examined tissue (Figure 4). No histological residues of CorMatrix were observed in the explanted tissue (several weeks after CorMatrix implantation). 

## Discussion 

The Kidney Disease Outcomes Quality Initiative guidelines suggest that the choice of material for an AVG should be based on the nephrologist’s or operator’s discretion and best clinical judgment, since the current evidence does not demonstrate that one graft material or modification thereof is associated with improved outcomes in terms of patency or complications [2]. PTFE has been the most commonly used material for almost 50 years and has served as the comparator or criterion standard for numerous studies [13]. 

Acellular extracellular bio-matrix scaffold CorMatrix is made from porcine intestinal submucosa and has been predominantly used for the reconstruction of cardiac and vascular tissue [5, 6, 7, 8] but can also be used in vascular access surgery instead of PTFE. 

Mild-to-moderate arm swelling is common after PTFE AVG placement, and it usually subsides quickly. This common perioperative complication was not observed in our group of patients with CorMatrix grafts. 

The risk of infection is more common in PTFE AVGs than in AVFs [14]. We observed no CorMatrix-related infections in our cohort. The low infection rate of CorMatrix is supported by a study by Shell et al. [15], who randomized 18 pigs into three groups (control group without bacterial inoculation, a group with bacteria-inoculated small intestine submucosa-derived extracellular matrix replacement of the common iliac artery, and a group of bacteria-inoculated PTFE vascular graft replacement of the common iliac artery). Grafts were explanted after 6 weeks, and showed no bacterial growth in quantitative cultures of the submucosa grafts, compared to PTFE grafts, which harbored bacterial colonies. 

The most common complication of PTFE AVGs is stenosis due to neointimal hyperplasia [4]. In our small case series, all the additional procedures to maintain or restore patency, including thrombendarteriectomy, percutaneous transluminal angioplasty with/without stenting, and surgical reanastomosis, were safe and feasible. 

This case series is the first to demonstrate the use of an acellular extracellular biomatrix scaffold for complete and partial reconstruction of hemodialysis vascular access. While prior reports have limited its use to AVF aneurysm repair or attenuation of venous neointimal hyperplasia, our experience shows that CorMatrix can be safely applied for all major vascular access constructions and reconstructions without significant perioperative or postoperative complications. However, in its current form, CorMatrix cannot be used routinely for AVGs due to the intraoperative suturing of longitudinal plates into tubular grafts. Therefore, pre-designed tubular grafts of different lengths and diameters (6 – 8 mm) are needed. 

The main limitation of our single-center study is the small number of patients. Further research, especially the development of pre-prepared tubular bio-grafts of varying diameters and lengths, is necessary for the broader use of resorbable extracellular bio-matrix in vascular access surgery. 

## Acknowledgments 

The authors wish to thank the Institute of Pathology, Faculty of Medicine, University of Ljubljana, Ljubljana, Slovenia, and the Hemodialysis and Internal Medicine Department at Trbovlje General Hospital for their support and cooperation in the vascular access program. 

## Authors’ contributions 

T.F. and B.L. conceived and designed the study. T.F. and N.K. collected the data, and N.K. performed the statistical analysis. N.K. wrote the manuscript. T.F. and B.L. read and critically evaluated the manuscript and, along with N.K., gave final approval for publication. 

## Funding 

The authors received no financial support for the research, authorship, and/or publication of this article. 

## Conflict of interest 

The authors declare no potential conflict of interest with respect to the research, authorship, and/or publication of this article. 

**Table 1.** Table 1. Surgical procedures with CorMatrix.

CorMatrix bridge graft between cephalic vein and lateral cephalic vein
De novo AVG
CorMatrix AVG between brachial artery and basilic vein (3 cases)
Reconstruction of high-flow AVF:
CorMatrix AVG between brachial artery and basilic vein (1 case)
CorMatrix AVG between brachial artery and cephalic vein (1 case)
CorMatrix as a reduction segment between cephalic and perforant vein → AVG thrombosis with reconstruction and extirpation of CorMatrix (1 case)
Reconstruction of aneurismatic AVF:
Aneurismoraphy with CorMatrix end-to-end anastomosis to fistula vein (3 cases)
AVF non-maturation due to radial artery thrombosis:
CorMatrix AVG between proximal radial artery and antebrachial vein (1 case)
Elongation of puncturing segment:
CorMatrix AVG between brachial artery and basilic vein (2 cases)
Elongation of CorMatrix AVG with new CorMatrix AVG (1 case)
Exposition/infection of PTFE graft
Reconstruction with PTFE extirpation and CorMatrix bridge graft (3 cases)
Degeneration of PTFE graft:
Complete removal of PTFE and replacement with CorMatrix graft (1 case)
RUDI:
Radial artery – CorMatrix – anterbrachial vein (3 cases)
Radial artery – CorMatrix – basilic vein (1 case)

AVF = arteriovenous fistula, AVG = arteriovenous graft, PTFE = polytetrafluoroethylene, RUDI = revision using distal inflow.
